# Pearls and Pitfalls of Weaning an Infant with Severe Atopic Dermatitis and Sensitization/Allergy to Food

**DOI:** 10.3390/jcm12123889

**Published:** 2023-06-07

**Authors:** Mattia Giovannini, Marta Bolis, Simona Barni, Giulia Liccioli, Lucrezia Sarti, Susanna Morelli, Matteo Pontone, Benedetta Pessina, Leonardo Tomei, Claudia Valleriani, Elio Novembre, Francesca Mori

**Affiliations:** 1 Allergy Unit, Meyer Children’s Hospital IRCCS, 50139 Florence, Italymatteo.pontone@unifi.it (M.P.);; 2 Department of Health Sciences, University of Florence, 50139 Florence, Italy; 3 Pediatrics Clinic, ASST-Spedali Civili of Brescia, 25123 Brescia, Italy; 4 Immunology Laboratory, Meyer Children’s Hospital IRCCS, 50139 Florence, Italy

**Keywords:** weaning, atopic dermatitis, sensitization, allergy, food, pediatrics

## Abstract

Atopic dermatitis (AD) is a common chronic inflammatory skin disorder in childhood. Skin barrier impairment exposes infants to food allergens, potentially causing sensitization followed by IgE-mediated food allergy. We describe the case of an infant with severe AD in whom several sensitizations to foods are detected, with consequently difficult weaning, and a history of anaphylaxis to cashew nut. Foods for which skin tests were negative were introduced into the infant’s diet. Then, when AD control was managed, oral food challenges (OFCs) for foods to which the patient was sensitized, with the exception of cashew nut, were performed. The simultaneous presence of sensitization toward multiple foods made it difficult to introduce them using classic OFC. Therefore, it was decided to perform the low-dose, gradual controlled OFC. This led to an introduction of sensitized foods into the infant’s diet, with the exception of cashew nut, avoiding allergic reactions. Absolute recommendations on how, when, and where to perform OFCs with allergenic food to which the child with AD is sensitized are lacking so far. In our opinion, OFCs and the subsequent ntroduction of allergenic foods should be individualized, evaluating some factors such as their social and nutritional importance, the patient’s age and clinical phenotype (including the history of anaphylaxis), and the sensitization profile. There is agreement on the fact that the dietary approach in children with moderate-severe AD should no longer include a strict elimination diet. We believe that an early, gradual controlled introduction of all allergenics to identify the amount of food tolerated in the absence of reactions, even if low dose, may improve patients’ and families’ quality of life. However, even if discussing a vast relevant literature, the limitation of our work is that we describe the management of a single patient. Extensive and high-quality research is needed in this field to improve the available evidence in the area.

## 1. Introduction

Atopic dermatitis (AD) is a common chronic inflammatory skin disorder in childhood, affecting up to 20% of children [1]. Food allergy (FA) is a frequent atopic disorder in the pediatric age group, affecting around 3% of children [2]. The great variability in FA prevalence among children may be related to geographical differences and different ways of diagnosing FA. For example, some studies include parents’ referred allergies, while others only evaluate test-diagnosed FA.

The genotype–phenotype association between AD and FA has been described in the literature [3,4]. In particular, both the severity and chronicity of AD increase the risk of FA development [5]. AD control is of paramount importance. The longer AD continues without correct management and the more severe AD is, the greater the probability becomes of a concomitant FA [6,7]. Infants and young children with AD are more commonly sensitized to food (e.g., egg) [8,9], while children over 5 years old are more commonly sensitized to aeroallergens (e.g., dust mite) [10,11,12,13]. In some cases, AD can occur as early as the first few months in the lives of exclusively breastfed infants.

Numerous studies have evaluated the role of breastfeeding in the development of AD. Indeed, some papers have found no association between breastfeeding and the development of AD [14,15,16]. Instead, other research favors a positive role of breast milk, with a decreased occurrence of AD [17,18,19,20]. Finally, a few studies associated breastfeeding with increased occurrence of AD [21,22] and food hypersensitivity clinical manifestations [23]. Indeed, to date, the results appear inconsistent. Therefore, further studies are needed to clarify the association between breastfeeding and the occurrence of AD.

On the other side, data in the literature regarding the role of moisturizer therapy in the risk of developing AD are currently inconclusive as well. More recent studies have shown that skin-barrier impairment exposes infants to food allergens and may be responsible, first, for sensitization and then, for IgE-mediated food allergies [24,25,26,27]. In some patients, food that a patient is sensitized to may exacerbate eczema flareups. Of note, there is no clear indication concerning early management of skin-barrier impairment. Indeed, it has been proved that application of moisturizers may be associated with a decrease in the development of sensitization and FA. However, it has also been reported that skincare through moisturization may contribute to the development of sensitization and FA [28]. Therefore, further research is also necessary to elucidate the relationship between moisturization and the development of sensitization and FA [29]. 

In contrast to delaying weaning, particularly with allergenic foods, the early introduction of certain allergenic foods is supported by the updated European Academy of Allergy and Clinical Immunology (EAACI) guidelines on the prevention of food allergies [30,31,32,33]. Peanuts and well-cooked eggs may be included in a complementary diet for a 4- to 6-month-old child.

In summary, what measures may help in preventing IgE-mediated FA and which children may need them the most is still a matter of debate. 

We describe the case of an infant with severe AD in whom several sensitizations to foods were detected with a consequent difficult weaning and a history of anaphylaxis to cashew [34,35]. 

## 2. Case Report

A 6-month-old infant exclusively breastfed and suffering from severe AD since he was 2.5 months was referred to the allergy unit of our hospital. He was sent for a clinical evaluation given the refractoriness of his skin lesions to optimal topical treatment with moisturizers plus corticosteroids and the need to start weaning. 

In his medical history, there was a positive family history of atopy (not specified) in the maternal line. Height and weight growth and psycho-physical development were in line with the standard of the age. First-level blood tests and allergy tests (skin prick test [SPT], prick-by-prick test [PbP], and specific immunoglobulins E [sIgE]) with common allergenic foods showed a polysensitization to cow’s milk and its fractions (casein, α-lactalbumin, and β-lactoglobulin), egg, and wheat (Table 1).

Due to severe skin involvement and sensitization to several allergenic foods, according to the results of the tests, it was suggested to continue exclusive breastfeeding by recommending an elimination diet for milk, egg, and wheat to the mother while breastfeeding and to wait to proceed with weaning for these foods. At the same time, AD treatment was optimized [36]. Furthermore, for the concomitant finding of severe hypereosinophilia and an increased level of total IgE (tIgE), to exclude an underlying cause, (e.g., immunological diseases and infectious diseases), in vitro tests (e.g., evaluation of immunoglobulins, lymphocyte subpopulations, and serologies for *Echinococcus* spp., *Toxocara* spp., *Strongyloides* spp.) and in vivo tests (abdominal ultrasound) were performed, all resulting negative [37].

Optimal topical skin treatment with steroids led to improvement of AD. While breastfeeding, AD showed an immediate worsening whenever the mother liberalized her diet and added even small quantities of the allergens to which he was sensitized, confirming the medical advice to avoid those allergenic foods in her diet.

However, at the age of 6 months, the patient’s weaning began. At first, foods for which SPT/PbP were negative (such as maize, rice, carrot, potato, rabbit, chicken, and turkey meat), were introduced into the diet with tolerance. The patient continued to show good height and weight growth and psycho-physical development. Peripheral eosinophil levels progressively became lower in parallel with better control of AD. At the age of 2 years, foods were tested again, and according to the results, the parents were advised not to feed the child with milk, nuts, egg, and wheat. All the other foods, including fish, were introduced to his diet at home in agreement with a dietician’s evaluation of the proper caloric intake and variety of his diet.

At the age of 2 years, the child had an episode of anaphylaxis after eating pasta seasoned with a pesto sauce (containing cashew nut) never eaten before. Particularly, he presented with swelling of lips and vomiting. He was treated at home with an oral steroid and immediately referred to a hospital. 

**Figure 1:**
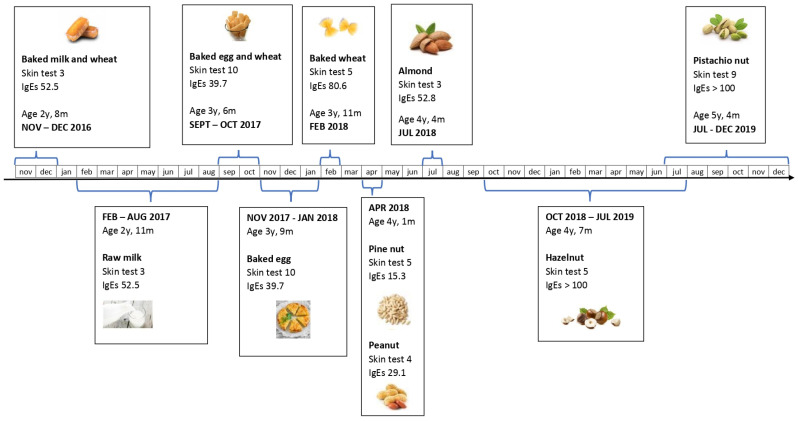
Open introduction into the diet of foods to which the patient was sensitized. Time elapsed between the first introduction and the liberalization of each food. Serving size used according to age and habits. Dose reached for the liberalization of each food (milk 134.6 mL, egg 35.6 g, wheat 41.2 g, pine nut 4.2 g, peanut 4.18 g, almond 3.78 g, hazelnut 8 g, and pistachio nut 4.1 g). sIgE: serum immunoglobulin E (kU/L), Jan: January, Feb. February, Mar: March, Apr: April, Jun: June, Jul: July, Aug: August, Sep: September, Oct: October, Nov: November, Dec: December.

At the age of 2 years and 8 months, for the improvement of AD control, the patient was subjected first to a low-dose oral food challenge (OFC), with baked cow’s milk within a wheat matrix, demonstrating tolerance. The procedure was therefore continued with the same and other foods in the following months, with a progressive and gradual controlled introduction of all foods to which the patient was sensitized (milk, egg, wheat, and nuts, with the exception of cashew nut, to which the patient manifested anaphylaxis) (Figure 1). Increasing doses of food proteins were tested in the hospital by several low-dose OFCs, and the regular administration of the dose tolerated for each food was continued at home. Once an age-adequate amount dose of the food was achieved, it was liberalized into the patient’s diet. 

Currently, the patient is 8 years old; he is affected by AD with an early onset and persistent phenotype associated with multiple sensitizations to food allergens and with a clinical history of food allergy for cashew nut. Furthermore, he has multiple sensitizations to inhalant allergens (e.g., dust mites, dog and cat epithelia, grasses, pellitory, hazel, and birch pollens) in the current absence of allergic respiratory clinical manifestations. The patient is on an elimination diet exclusively for cashew nuts, and he continues with regular applications of emollients and as-needed topical corticosteroids (although he less frequently flares up with skin lesions), with good control of AD.

## 3. Discussion

We presented the clinical case of an exclusively breastfed infant affected by early onset AD persistent throughout his life so far, associated with elevated tIgE values, severe hypereosinophilia, and multiple sensitizations to food antigens before starting weaning. It is known that food sensitization and FA may improve or solve with age, but they may also persist. Hence, adequate investigation and management are needed. The management of such a case is very complex and requires the collaboration of several specialists (e.g., the allergist, the immunologist, the dermatologist, and the dietician).

The finding of high tIgE values and severe hypereosinophilia in our case required the execution of tests to exclude other possible disorders (e.g., immunological diseases and infectious diseases). In our case, the needed tests were carried out, and the results were negative.

Optimal management of AD requires optimal topical treatment to obtain good control of the skin lesions and the potential evaluation of possible concomitant sensitization (e.g., to foods and inhalants) in selected cases. In this context, for example, according to the EAACI recommendations [38], in patients with persistent moderate-severe AD, if the food has not yet been ingested or regularly ingested in an adequate quantity, in vivo skin tests and in vitro (tIgE, sIgE) allergy tests to detect specific sensitizations against common food allergens are recommended, even in the absence of a history of immediate reactions to food. It has been proved that in patients with AD, the percentage of food sensitization is variable, but the percentage of confirmed FA is much lower [39,40,41]. For this reason, the OFC remains the gold standard for the diagnosis of an FA.

Indeed, it is mandatory to distinguish that sensitization to one or more allergens is different from an allergy [2]. Screening for FAs may prevent morbidity for food allergy in patients affected by AD. On the other side, it may result in avoidance of the food to which testing is positive in sensitized but not allergic patients. Of note, considered that early introduction of some food allergens may reduce the risk of FA, unnecessarily avoiding a food based on the results of testing alone in sensitized but not allergic patients may result in the development of FA because that food is avoided.

Hill et al. [42] evaluated a subgroup of infants with moderate–severe AD, showing that 90% of these patients have sIgE sensitization to at least one food. Eigenmann et al. [43] proved that nearly 40% of children with moderate–severe AD has clinically significant IgE-mediated food allergies. Overall, the earlier the onset and the more severe the AD is, the more likely the infant may develop a FA [44].

The observational cohort study conducted by Roduit et al. [45] wanted to better define the association between particular AD phenotypes and the phenomenon defined as atopic march (progression to other atopic disorders). This study showed that the persistent early AD phenotype (with onset within 2 years of age and persistence of signs and symptoms up to the age of 6) is strongly associated with sensitization to food allergens, with positive familial history of atopy, with higher SCORAD, and with increased risk of developing FA, rhino-conjunctivitis, and asthma.

Paller et al. [46] defined risk factors responsible for increasing the risk of AD progression to other atopic disorders: polysensitization, AD persistence, early age of onset, greater skin disease severity, parental atopy and having a filaggrin (FLG) gene mutation.

The case reported in this paper presents the characteristics of the persistent early phenotype described by Roduit et al. and the risk factors associated with the development of allergic march described by Paller et al., with the exception of mutation of the FLG gene (which has not been performed).

Due to the severity of the skin disease, the flareups of AD with the mother’s unrestricted diet when she added even small quantities of allergens to which he was sensitized, we recommended the abovementioned elimination diet to her while continuing breastfeeding [47]. Our patient continued with exclusive breastfeeding until 6 months of age, when weaning began by introducing into his diet foods for which SPT/PbP were negative. The introduction of other foods to which the child was sensitized was instead started at the age of 2 years and 8 months, once AD was well-controlled. Potential allergenic foods were introduced after achieving AD control because AD severely influenced the patient’s life, and his parents refused to expose him to the risk of adverse reactions to OFC during this period.

In the last decades, various strategies have been proposed for introducing complementary nutrition to prevent FA, and there has been a change in course in international guidelines from delayed weaning to early weaning. In fact, while in the 1980s and 1990s, it was suggested that early exposure to solid foods may be associated with the development of allergic disease [48,49,50], later studies have shown that the avoidance strategy was not effective, suggesting, conversely, that oral tolerance may be induced by allergen exposure rather than allergen avoidance [51,52]. However, the American Academy of Pediatrics (AAP) already updated its previous recommendations in 2008, underlining the absence of sufficient evidence to recommend maternal elimination diets, as well as the delayed introduction of potentially allergenic foods into the infant’s diet, for the prevention of FA [53,54].

All this led them to abandon the allergenic avoidance strategy and led various groups to perform several randomized controlled trials to evaluate if the early introduction of peanuts, eggs, and cow’s milk into the diet of high-risk infants or the general population may reduce the risk of developing an allergy to these foods (e.g., Learning Early About Peanut allergy (LEAP) [31], Enquiring About Tolerance (EAT) [55], and Prevention of Egg Allergy with Tiny Amount Intake (PETIT) [33] studies). With the modification of the evidence, recommendations suggested by the most important international guidelines (American Academy of Allergy, Asthma and Immunology (AAAAI) [56], EAACI [30], Australasian Society of Clinical Immunology and Allergy (ASCIA) [57], and Asia Pacific Academy of Paediatric Allergy, Respirology and Immunology (APAPARI) [58]) regarding the introduction modalities of complementary foods into the diet for prevention of food allergies, primarily in the high-risk population (e.g., in patients with AD and/or food sensitization), have therefore changed. However, convincing data about the efficacy of early introduction of allergenic foods are available only for eggs and peanuts [32,59,60]. Indeed, so far, there is no evidence to support the early introduction of other allergens (e.g., milk, wheat, fish, and sesame) to prevent FA development [32].

In summary, on one side, sensitization to multiple allergens in children with AD may be not searched because it is often clinically irrelevant and not beneficial from the cost-effectiveness point of view. On the other side, specific food allergens should be tested in case of moderate–severe AD. In the case of detection of sensitization toward one of these allergenic foods, potential tolerance should be checked with an OFC promptly to rule out a possible clinically irrelevant sensitization before a long unneeded exclusion diet [61,62,63,64,65].

So far, the severity of reactions during OFC cannot be predicted on the basis of the level of sIgE or the size of skin tests with foods. In any event, a positive OFC is usually expected when the patient has a high level of sIgE or size of skin test for the food suspected [66].

In our case, the simultaneous presence of sensitization toward multiple foods has made it difficult to introduce foods with a classic OFC because of the risk of possible allergic reactions. In fact, due to the high values of size of skin tests and level of sIgE for some foods, it was decided to split the doses and not reach the total dose in a single session but in a fractionated way with several low-dose OFCs at intervals of 2–3 months, performing a gradual controlled OFC [67,68]. The acquisition of tolerance for each introduced food was, thus, favored in the absence of allergic reactions, allowing the progressive liberalization of the foods themselves in the diet (with the exception of cashew, to which the patient manifested anaphylaxis).

On the other side, we cannot exclude that tolerance toward some sensitizing foods (such as milk and egg) may reflect the already known natural course of FA in children: about 80% of children acquire spontaneous tolerance toward milk and egg by the age of 6 years [69].

Multiple issues influenced the management of our case, including the great number of sensitized foods, the high level of sIgE, the target of a good quality of life for our patient, and the conciliation with the working needs of his parents. All these issues led us to adopt tailored management based on progressive low-dose OFCs and liberalization of tested foods.

In fact, according to Japanese guidelines, the objective of the OFC is not only to demonstrate the allergy to a specific food but also to identify the quantity of food tolerated in the absence of reactions, to implement the “minimum elimination diet” [70]. This means that a positive OFC may not always imply the need for complete elimination of the food from the diet. Even in the case of a positive OFC, the patient may be instructed to consume at home even small amounts of the food or its hypoallergenic form (e.g., baked), if tolerated, taking into consideration potential cofactors, such as physical exercise [71]. However, a strict diet should be recommended for children who show reactions, e.g., that are severe, at very low doses of allergens during OFC or in the presence of cofactors such as physical exercise. In this case, the low-dose, gradual controlled OFC performed in the hospital was the right option associated with the simultaneous treatment of AD and the nutritional evaluation of the patient.

Finally, the temporal order in which foods with positive skin tests can be introduced into the diet of patients can be decided through shared decision-making with the parents by considering several factors, including their nutritional and social importance, the skin tests and sIgE values, the patient’s clinical history (sensitization or allergy), and the patient’s age. In our specific case, we decided, also in accordance with the dietary service of our hospital, to start with nutritionally fundamental foods such as milk, egg, and wheat. The introduction of these foods may be undertaken, in general, as soon as the skin condition makes it possible, when the child is ready for the process.

## 4. Conclusions

Absolute recommendations on how, when, and where to perform OFCs with allergenic food to which the child with AD is sensitized are lacking so far. In our opinion, OFCs and the subsequent introduction of allergenic foods should be individualized, evaluating some factors such as their social and nutritional importance, the patient’s age and clinical phenotype (including the history of anaphylaxis), and the sensitization profile.

There is agreement on the fact that the dietary approach in children with moderate–severe AD should no longer include a strict elimination diet. We think that an early, gradual controlled introduction of all allergenics to identify the amount of food tolerated in the absence of reactions, even if low dose, may improve patients’ and families’ quality of life. However, even if discussing a vast relevant literature, the limitation of our work is that we describe the management of a single patient. Extensive and high-quality research is needed to improve the available evidence in the area.

## Figures and Tables

**Table 1:** SPT, PbP, and sIgE for milk, egg, wheat, and nuts with increasing patent’s age. Tests for nuts were not performed until 2 years of age since they are not usually part of Italian children’s diet until that age. Of note, SPT was performed for egg white and wheat, while PpP was performed for milk, nuts, and fish. SPT: skin prick test; PBP: prick-by-prick test; tIgE: total immunoglobulin E; sIgE: specific immunoglobulin E; np: not performed; -: negative.

AGE	6 Months		2 Years		4 Years	
tIgE (kU/L)	1777		2863		4155	
Eosinophils (n °/mmc)	6087		708		378	
Foods	SPT/PbP (mm)	sIgE (kU/L)	SPT/PbP (mm)	sIgE (kU/L)	SPT/PbP (mm)	SIgE (kU/L)
MILK	6	47.60	3	52.50	-	17.00
EGG WHITE	4	>100.00	10	39.70	-	19.10
WHEAT	4	>100.00	5	80.60	-	41.10
PEANUT	np	np	2	19.30	4	29.10
PISTACHIO	np	np	7	>100.00	9	>100.00
CASHEW	np	np	7	>100.00	10	>100.00
HAZELNUT	np	np	3	>100.00	5	>100.00
ALMOND	np	np	2	>100.00	3	52.80
WALNUT	np	np	0	14.40	4	26.00
PINE NUT	np	np	0	3.81	5	15.30
FISH	-	-	-	-	-	-

## Data Availability

Not applicable.

## Abbreviations

AAAAI = American Academy of Allergy, Asthma & Immunology; AAP = American Academy of Pediatrics; AD = Atopic Dermatitis; APAPARI = Asia Pacific Academy of Pediatric Allergy, Respirology & Immunology; ASCIA = Australasian Society of Clinical Immunology and Allergy; EASI = Eczema Area and Severity Index; EAT = Enquiring About Tolerance; EAACI = European Academy of Allergy and Clinical Immunology; FA = Food Allergy; LEAP = Learning Early About Peanut allergy; OFC = Oral Food Challenge; PETIT = Prevention of Egg Allergy with Tiny Amount Intake (PETIT); PbP = Prick-by-Prick test; POEM = Patient-Oriented Eczema Measure; SCORAD = SCORing Atopic dermatitis; SPT = Skin Prick Test; sIgE = Specific Immunoglobulin E; tIgE = Total Immunoglobulin E.
